# A numerical study of pre-polarisation switching in ultra-low field magnetic resonance imaging using dynamic permanent magnet arrays

**DOI:** 10.1038/s41598-020-74931-y

**Published:** 2020-10-23

**Authors:** Ruben Pellicer-Guridi, Michael W. Vogel, Viktor Vegh, Jiasheng Su, Matthew S. Rosen, David C. Reutens

**Affiliations:** 1 Centre for Advanced Imaging, The University of Queensland, Brisbane, QLD Australia; 2 A. A. Martinos Center for Biomedical Imaging, Charlestown, MA USA; 3 Harvard Medical School, Boston, MA USA; 4 Department of Physics, Harvard University, Cambridge, MA USA; 5 Australian Research Council Training Centre for Innovation in Biomedical Imaging Technology, The University of Queensland, Brisbane, QLD Australia

**Keywords:** Imaging techniques, NMR spectroscopy, Magnetic resonance imaging, Three-dimensional imaging, Biomedical engineering, Mechanical engineering, Computational science

## Abstract

Dynamically adjustable permanent magnet arrays have been proposed to generate switchable magnetic fields for pre-polarisation in Ultra-Low Field magnetic resonance imaging. However, the optimal switching dynamics of the pre-polarisation magnetic field as well as the energy requirements, mechanical forces and stresses during switching of the pre-polarisation field have not been evaluated. We analysed these requirements numerically and estimated the magnetic resonance signal strength and image quality for two practical switching modes in an instrument suitable for scanning the human head. Von Mises stress analysis showed that although magnetic forces were significantly higher for two specific rungs, the structural integrity of magnet rungs would not be compromised. Our simulations suggest that a significantly higher signal yield is obtained by switching off the pre-polarisation field with the angular velocity in each rung dependent on its location.

## Introduction

Permanent magnets have become a reliable and powerful magnetic field source without current flow^1^. Rare earth magnets, such as Neodymium magnets, can generate strong magnetic fields with exceptionally high temporal stability^1,2^ and are suited to applications in low field magnetic resonance imaging (MRI) in which high quality magnetic fields with field strengths between 0.1 and 0.5 T in a large field of view (FOV) are required. The benefits of strong permanent magnets are also exploited in ultra-low field (ULF) MRI systems (readout field < 10 mT) for sample pre-polarisation^3–5^. Previously described pre-polarisation techniques entail the temporary placement of the sample within a strong homogeneous magnetic field to increase its net magnetisation, followed by rapid translation of the sample into a weak magnetic field for signal acquisition. Such an approach is not feasible for human imaging due to the accelerations involved in moving the sample between high and low field locations^5,6^. Current human ULF MRI devices generate pre-polarisation fields by switching a strong current through resistive coils^7–9^. Such electromagnets considerably increase the power requirements of the system, limiting its portability.

**Figure 1:**
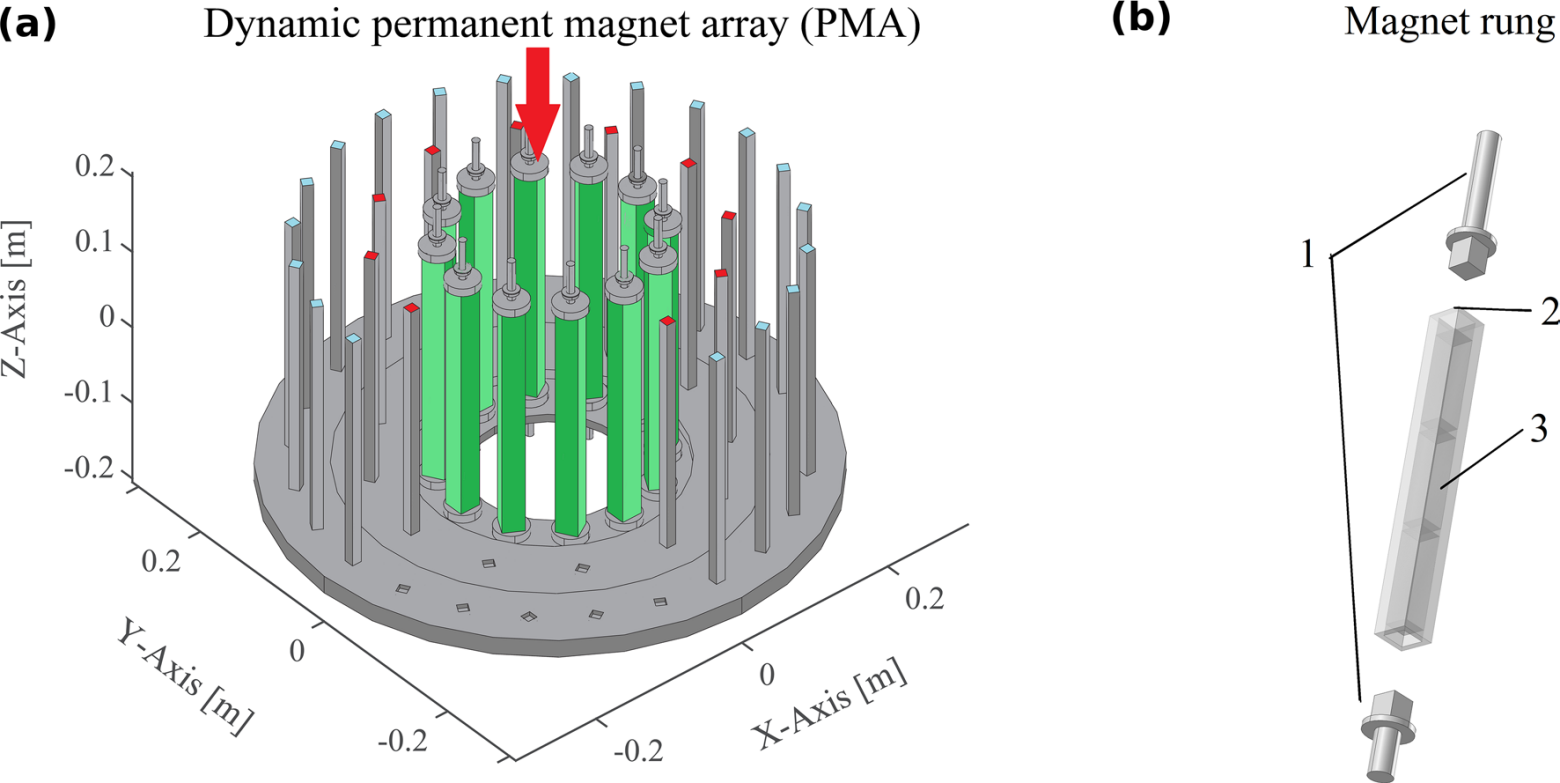
Representation of the proposed dynamic permanent magnet array and the composition of a pre-polarisation magnet rung. (**a**) Compact 3D ULF-MRI model with three nested cylindrical permanent magnet arrays. The inner array (green colour) comprises rotatable permanent magnet rungs for switching pre-polarisation B_p_. The two outer permanent magnet arrays, each with the Halbach magnetisation pattern (blue and red top colour), control the constant measurement field **B**_**m**_. In this illustration, some front magnets, the encoding magnet array and the head phantom are not shown to allow the inner array to be viewed. (**b**) Exploded view of a rectangular magnet rung of the inner array. It is terminated on each side by two flanges (part 1) that are fitted into bearings that allow free rotation about its symmetry axis and consists of a fibreglass reinforced polyester (FRP) tube (part 2) with three individual Neodymium magnets (part 3) inside.

Recently, we described dynamically adjustable cylindrical small permanent magnet arrays (Fig. 1a) to generate the magnetic fields required in ULF MRI. In this concept, we introduced the possibility of switching off the magnetic field from a cylindrical Halbach array enabling permanent magnets to be used to generate the pre-polarisation field. The switching is achieved through magnet rungs with freedom of rotation for each rung along its own axis. This work proposed that realigning the permanent magnets from a Halbach configuration to a tangential configuration resulted in near magnetic field cancellation^10^. In that work we built static prototypes to validate the “on” and “off” states of the pre-polarisation array. However, the feasibility of the dynamics of the switching transition and its mechanical requirements were not analysed. Given the fast switching times required to turn off the pre-polarisation field (< 100 ms)^11^ a numerical study to verify the feasibility of constructing such a mechanism is needed. Furthermore, during switching, magnet rotation results in temporal evolution and spatial variation in the magnitude, homogeneity and directionality of the magnetic field. The effects of magnetic field evolution during pre-polarisation switching and its effect on signal generation and image quality are yet to be evaluated. In this paper, we undertake these evaluations as they are critical for the understanding of the mechanical feasibility of the actuators and the efficiency of pre-polarisation. The studies described here deal with an ULF MRI instrument suitable for imaging the human head based on small permanent magnet arrays. We focus on two switching modes in a circular magnetic array, (a) one in which each magnet rung rotates with equal angular velocity, so that rotation stops at different times for different magnet rungs, and (b) one in which angular velocity varies with the location of the magnet in the array such that rotation of all magnets stops simultaneously. The effects of the switching modes on the temporal evolution and spatial distribution of the magnetic field, energy and force distribution, and generated MRI signal for a 3D digital Shepp-Logan phantom were evaluated. We also analysed the mechanical strains induced by the static magnetic forces and torques during acceleration/deceleration.

## Materials and methods

### Permanent magnet rung array design

Figure 1a shows the permanent magnet rung array for the ULF MRI instrument developed at the Centre for Advanced Imaging, The University of Queensland. The permanent magnet array responsible for the pre-polarisation field (**B**_**p**_ = 48 mT) is shown in green. Two outer nested permanent magnet arrays with the static Halbach magnetisation pattern generate the variable measurement field (**B**_**m**_, Fig. 1a). For simplicity, we have omitted the single permanent magnet required to create the encoding magnetic field (**B**_**enc**_)^12^. **B**_**m**_ and **B**_**enc**_ are of significantly lower magnitude than **B**_**p**_ and were excluded in the force and energy analysis. However, to simulate a realistic MRI signal, the measurement arrays were included in the COMSOL model to calculate an accurate **B**_**m**_ distribution.

We evaluated the magnet field evolution and mechanical forces and stress distribution for permanent magnet arrays (Fig. 1a) with a radius *r*_*A*_ = 0.18 m and a total height *h*_*a*_ = 0.3 m. The analyses of image quality and magnetic field evolution were performed for an array with 12 magnet rungs (see Fig. 1b). To evaluate the effect of magnet number on magnetic forces and torques, comparisons were made between arrays with 12, 16 and 20 rungs.

**Figure 2:**
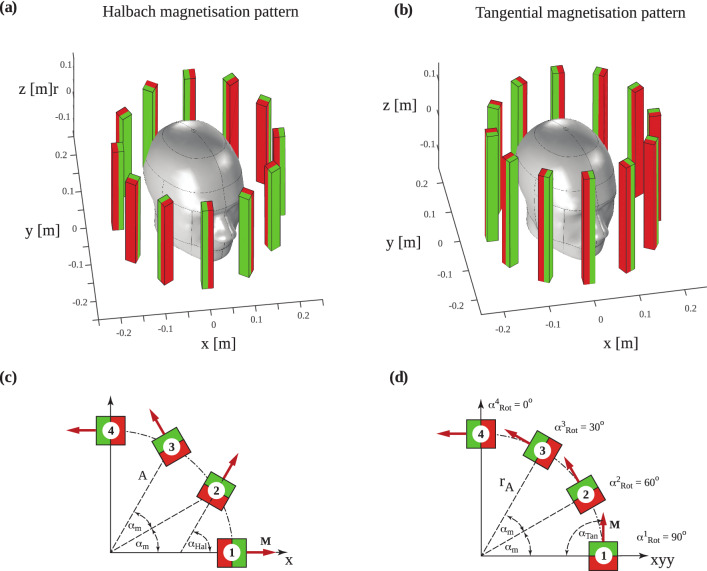
Array with 12 permanent magnet rungs and their orientation for the pre-polarisation on and off positions. Rungs are placed equidistantly along the circumference around a human head. The magnetic North is coloured green and the magnetic South is coloured red with the magnetisation (**M)** direction pointing from South to North. (**a**) Rungs form the Halbach magnetisation pattern to switch on pre-polarisation. (**b**) Rungs form the tangential magnetisation pattern to switch off pre-polarisation. (**c**) Sectional transverse view of four rungs in the Halbach magnetisation pattern to illustrate the array radius r_A_, magnet orientation angle *α*_m_ and magnetisation orientation angle *α*_Hal_ with respect to the positive x-axis. The magnet rungs are numbered counter-clockwise starting from the positive x-axis. (**d**) Sectional transverse view of four rungs in the tangential magnetisation pattern. The calculated rotation angles *α*^n^_Rot_ (n = 1, 2, 3 and 4) between the Halbach orientation angle *α*_Hal_ and the tangential orientation angle *α*_Tan_ are shown. The positive sign indicates counter clockwise rotation about the z-axis.

Each rung comprises a square tube of fibreglass reinforced polyester (Part 1, Fig. 1b), an electrical insulator known for its light weight, high bulk strength and stiffness, enclosing three commercially available Neodymium magnets sized 2.54 cm × 2.54 cm × 10 cm (part 2, Fig. 1b). The remanent magnetisation (**B**_**r**_) of each magnet was 1.45 T, oriented in the transverse (xy-) plane and perpendicular to the long axis of the magnet as illustrated in Fig. 2. Individual magnets are not bonded together. Appropriate bearings enable rotation about the z-axis of each rung. We assume that the shafts are fitted in high quality, low resistance bearings allowing us to neglect friction and any transverse (xy-plane, see Fig. 9b,c) displacement. We note that any permanent magnet shape can be readily accommodated in our simulation and analysis.

### Simulation environment

We employed COMSOL (version 5.0, AC/DC module, magneto-static and structural mechanics, COMSOL AB, Stockholm, Sweden) for the numerical analysis. The array model (see Fig. 1a) was discretised in 3D-tetrahedral meshes using predetermined mesh distributions and densities based on COMSOL’s recommendations. Manual mesh density enhancement was used for surface integration of the Maxwell stress tensor to ensure accurate and convergent results when calculating magnetic forces and torques. For additional numerical stability, each magnet rung was modelled with small chamfers (1 mm) along each edge. The cylindrical computational window, 1.2 m in both diameter and length, was large enough to encompass the array and minimise numerical errors due to boundary effects. The relative magnetic permeability of Neodymium was set at 1.05^13^ and that of the fibreglass tube and the surrounding environment was set at 1. We did not include the support structures and frames in the COMSOL model for simplification since they are made of non-magnetic materials. However, we accurately modelled a single magnet rung with appropriate boundary conditions to mimic the external frame to ensure that our evaluation of maximal mechanical stress during switching was realistic.

### Magnetisation pattern and switching mode

**Figure 3:**
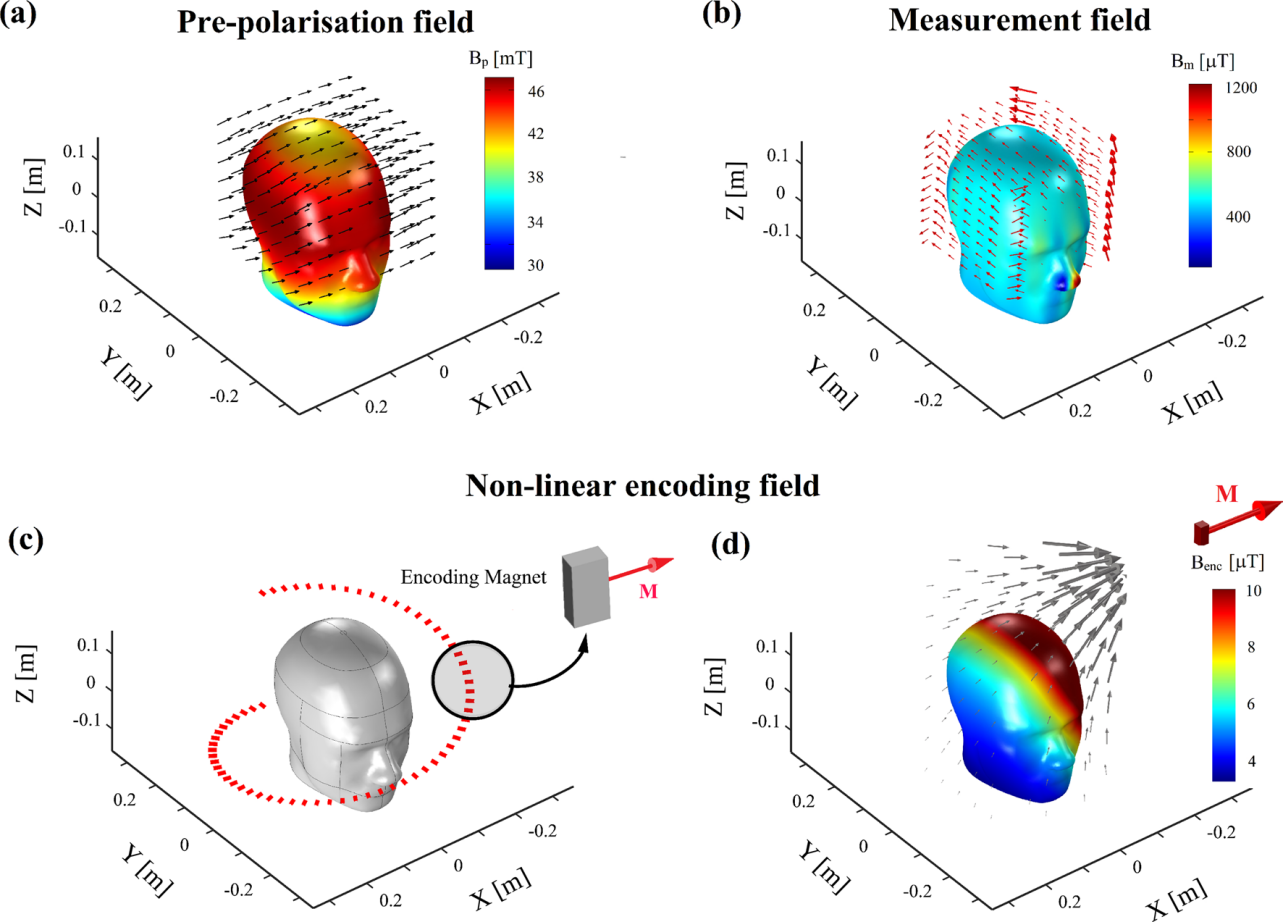
Magnetic fields required for ULF-MRI. (**a**) Sample pre-polarisation field **B**_**p**_ generated by the pre-polarisation magnet array in the Halbach magnetisation pattern. (**b**) Measurement field **B**_**m**_ generated by the two outer nested Halbach dipole arrays and the pre-polarisation array in the tangential magnetisation pattern. (**c**) Principal encoding magnet with helical path around the head phantom. Each line segment corresponds to the encoding magnet location and magnetisation direction. (**d**) Encoding field generated at one location by the small magnet. The magnetic field distribution corresponds to a magnetic dipole because of the relatively small magnet dimension.

**B**_**p**_ is generated by forming the Halbach magnetization pattern in the cylindrical array (Fig. 2a). **B**_**m**_ is generated by two nested cylindrical static Halbach dipole arrays (see Fig. 1a) when the pre-polarisation array forms a tangential magnetization pattern (Fig. 2b) to switch off **B**_**p**_^10^. Figure 3 displays each magnet rung symbolically in red and green to illustrate the magnetic South and North Poles, respectively. By convention, the magnetisation vector **M** points from the South Pole to the North Pole (Fig. 2c,d). For the Halbach magnetisation pattern, the orientation angle *α*_*Hal*_ of each rung (Fig. 2c) is described by

1 $$ \alpha_{Hal}^{n} = 2 \cdot \alpha_{m} \left( {n - 1} \right) $$

with *n* being the rung number, *n*_*tot*_ the total number of permanent magnet rungs and *α*_*m*_ = 2π/*n*_*tot*_. With this representation, **B**_**p**_ aligns with the x-axis. The rung orientation angles *α*_*Tan*_ (Fig. 2d) for the tangential magnetisation pattern are described by

2 $$ \alpha_{tan}^{n} = \alpha_{m} \left( {n - 1} \right) + \pi /2. $$

For each rung, the rotation angle required to switch between the two patterns is obtained by

3 $$ \alpha_{rot}^{n} = \alpha_{m} \left( {n - 1} \right) - \pi /2. $$

To analyse the switching from the Halbach to the tangential magnetisation pattern, time discretisation was into 13 uniform steps from *t*_0_ (Halbach magnetisation pattern i.e. ‘on’) to *t*_12_ (tangential magnetisation pattern i.e. ‘off’) and considered two switching modes as noted above. According to Eq. (3) each magnet rung requires different rotation angles *α*_*rot*_. In the first switching mode, angular velocity is uniform between rungs and rotation stops at different times for each rung *t*_*k*_ (k = 0, 1, 2,··12) depending on its location. For the second switching mode, all rungs stop rotating at *t*_*12*_. We enforced a maximum rotation angle *α*_*rot*_ ≤ π by reversing the rotation direction for magnet rungs with *α*_*rot*_ ≥ π according to Eq. (3) to ensure the most rapid pre-polarisation field switching. For a 12 magnet array (*α*_*m*_ = π/6), the maximum rotation angles for magnets 11 and 12 (see Fig. 2d) are *α*^*11*^_*rot*_ = 7/6π and *α*^*12*^_*rot*_ = 8/6π, respectively. Hence, magnets 11 and 12 counter rotate by *α*^*11*^_*rot*_ = − 5/6π and *α*^*12*^_*rot*_ = − 4/6π.

### Forces and mechanical stress in permanent magnet rungs

The magnetic attracting or repelling forces were calculated by integrating the Maxwell stress tensor over the magnet surface^14^. Torsional or shear stress and bending in the magnet rungs is induced by external torques due both to inertia during rapid angular acceleration/deceleration and to the repelling/attracting magnetic forces. This stress results in shape deformation which might cause damage (fracture or yield). We applied the Von Mises yield criterion in COMSOL, which indicates the level at which a ductile material yields or fractures under load, and compared it with the shear modulus *G* of the Neodymium magnet. We assumed a safety factor of 10, i.e., a maximal Von Mises stress one order of magnitude below *G*. This stringent condition is an efficient engineering approach for prototyping as it allowed us to perform combined simulation with the materials used here, i.e., permanent magnets, fibre-class tubes and brass holders. It should be noted that, when refining materials for mass production, more appropriate criteria for brittle materials could be used like the Rankine, Coulomb or Mohr–Coulomb criteria^15^. *G* was calculated using known Young’s modulus *E* and Poisson’s ratio: *G* = *E*/2(1 + υ). Table 1 lists the material parameters required for the simulation. Based on the simulation results shown in the mechanical stress section, we assumed that the strongest magnetic force **F**_**mag**_ is perpendicular to the magnet surface and that the torques are applied at both ends and in opposite directions. Since frictionless rotation was assumed due to the use of high quality bearings, the applied torque τ_**act**_ transmitted from the external actuator system is counterbalanced only by the tangential component of the magnetic force (surface normal **n** ⊥ **F**_**mag**_) for the magnet rung at rest and (b) the torque τ_**in**_ during acceleration due to inertia.

**Table 1:** Material properties employed in COMSOL for mechanical stress analysis.

	SI-unit	Neodymium	Fiberglass (FRP)	Shaft (brass)
Density	g/cm^3^	7.40	2.1	8.49
Young's modulus E	N/m^2^	1.6 × 10^11^	4.30 × 10^10^	97 × 10^11^
Shear modulus G	N/m^2^	6.452 × 10^10^	1.72 × 10^10^	37 × 10^11^
Relative magnetic permeability µ_r_	1	1.05	1	1
Poisson's Ratio υ	1	0.24	0.24	0.31

### Signal generation and imaging

Figure 3 illustrates the magnetic fields generated for imaging. The pre-polarisation field **B**_**p**_ for sample magnetisation is generated by the pre-polarisation array with the Halbach magnetisation pattern (Fig. 3a). After **B**_**p**_ is rapidly (≈ 10 ms) switched off, the magnetisation vectors **M** precess about **B**_**m**_ applied along the y-axis (Fig. 3b), with the Larmor frequency determined by the magnitude of **B**_**m**_^10,16–18^. Rapid switching ensures that the non-adiabatic condition is valid allowing imaging without additional RF pulses^16^. **B**_**m**_ is static and of much lower magnitude than **B**_**p**_. Changes in **B**_**enc**_ form the basis for spatial encoding and image acquisition. We considered **B**_**enc**_ produced by a single magnet moving around the head phantom on a helical path in one revolution as shown in Fig. 3c. Since the encoding magnet is much smaller than the distance to the sample, the magnetic field distribution corresponds to that of a magnetic dipole allowing **B**_**enc**_ to be calculated analytically. As visualised in the example of Fig. 3d, **B**_**enc**_ is spatially non-linear, necessitating the application of back-projection based methods for reconstruction^12^. The spatially dependent sample magnetic property, **m,** is related to the measured time dependent signal *s(t)* via an encoding matrix **E**_**en**_ by

4 $$ s(t) = {\text{E}}_{{{\text{en}}}} (q,t){\text{m}}(q), $$

where *q* denotes spatial location. Each line of **E**_**en**_ corresponds to the magnetic field distribution within the entire discretised field of view with each matrix element describing the local phase accumulation of the spin precession at one point. In this study, we simulated encoding using one helical path over the whole array length.

To simulate the signal in a single receiver coil generated by the spin evolution of **M** as described by Bloch’s equation, we superposed the contribution of individual spins located at *q*^19^. This is feasible since the only relevant spin–spin interactions at this field strength are described by the measured relaxation times T_1_ and T_2_^20^. At each sample point *q*, we evaluated the magnetic field in COMSOL and imported the data into MATLAB programs (MathWorks, Natick, MA, USA) developed in our laboratory to simulate signals and image reconstruction. All COMSOL simulations were carried out using an × 64-based 16 core PC with 128 GB of RAM, while the MATLAB simulations were run on an × 64-based 8 core PC (DELL Optiplex 9020) with 64 GB of RAM.

In the imaging studies, we implemented a 3D digital phantom based on the Shepp-Logan phantom, assuming realistic human brain tissue relaxation times of T_1_ = 100 ms and T_2_ = 80 ms^7,21^ for all compartments. Proton densities ranging from 1 to 70% (where 100% represents pure water) were assigned to different compartments^22^. We used the same T_1_ and T_2_ for different compartments as the comparison of different switching approaches was our primary goal. Noise was added to the simulated MRI signals to visualise the efficiency of both switching methods. The amplitude of the noise was approximated to SNR levels achievable with room temperature ULF surface coils (SNR ≈ 30)^23^. We centred the phantom within the field of view (FOV; dimensions: 0.18 m × 0.18 m × 0.18 m) discretised isotropically into 32 × 32 × 32 points. The quality of the reconstructed images was quantified by performing a voxel-wise subtraction between normalised original and reconstructed images, and averaging the difference across all voxels.

## Results

### Magnetic field distribution

**Figure 4:**
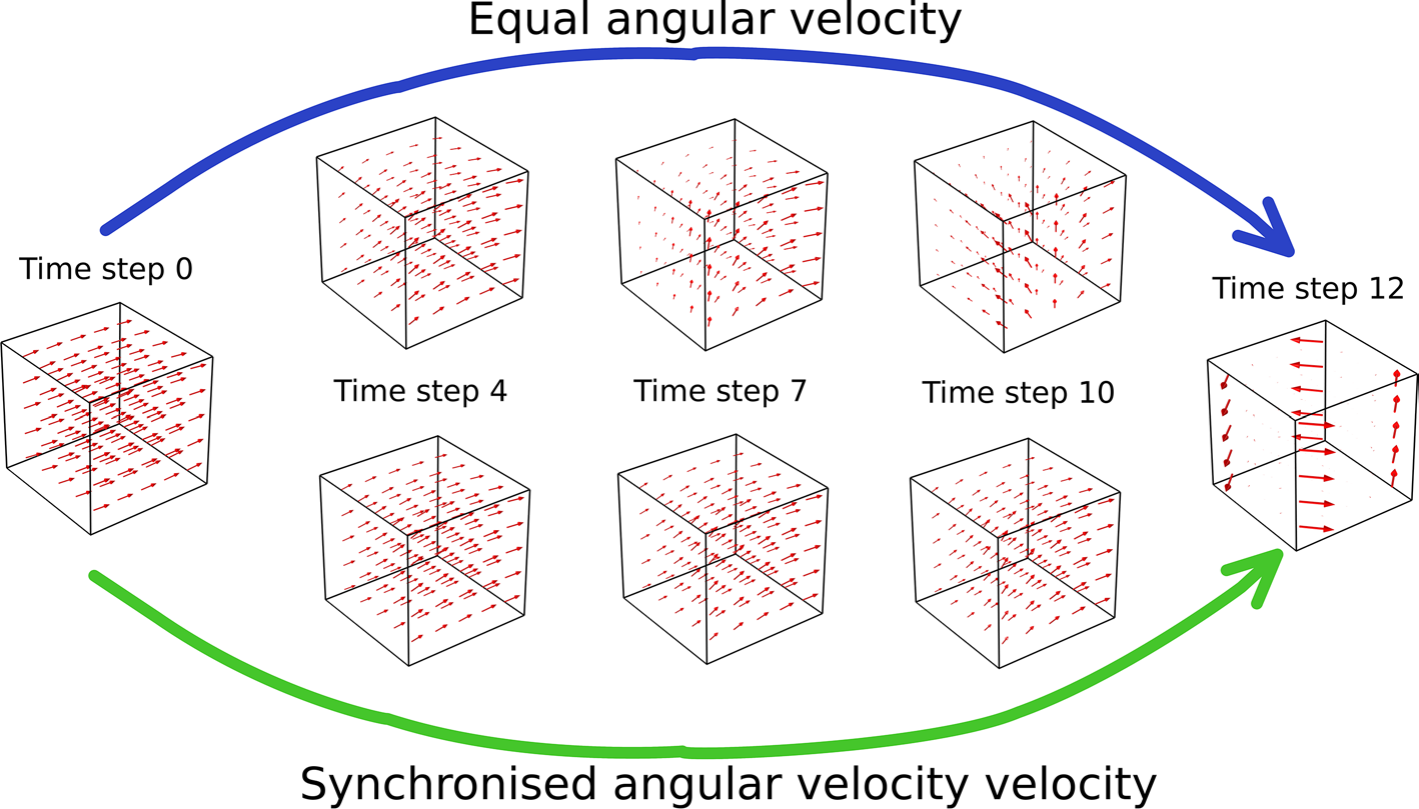
COMSOL magnetic field vector plot within the cubic field-of-view for a sequence of time steps. From left to right, time steps varying from t_0_ (switched on) to time step t_12_ (switched off). Two switching modes are shown: Equal angular velocity for all magnets (top row) and synchronised velocity (bottom row). For the purposes of illustration, the phantom has been removed.

Figure 4 illustrates the magnetic field distribution as a volume arrow plot in a cubic field of view located at the centre of the pre-polarisation array. The fields for 5 time steps, *t*_0_ (**B**_**p**_ switched on), *t*_4_, *t*_7,_ *t*_10_ and *t*_12_ (**B**_**p**_ switched off) are shown. Magnet rung rotation with equal angular velocity is shown on the top row and switching with location-dependent angular velocity is shown in the bottom row. For the purposes of illustration, vector plots at *t*_12_ have been scaled up to visualise the weak magnetic field (< 10 µT) compared to the magnitudes at the other time steps. For equal angular velocities, the magnetic field quickly disperses after time step *t*_4_ and changes direction, while for the location-dependent rotation speeds, field homogeneity and directionality were preserved for a longer time (until time step *t*_10_).

### MRI signal strength and image reconstruction

**Figure 5:**
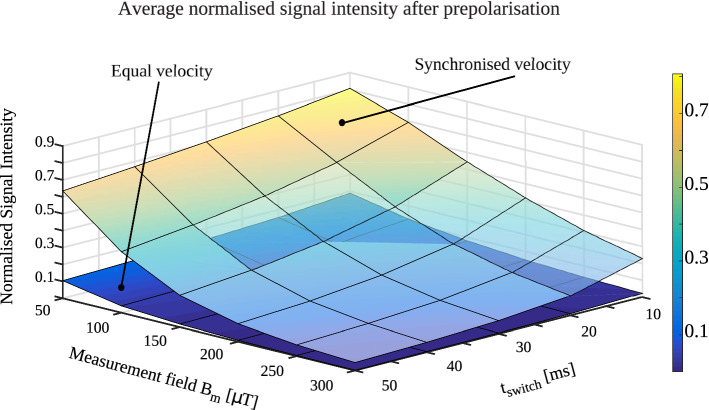
3D surface plot of the normalised MRI signal strength after the pre-polarisation magnetic field is switched off. The signal yield, evaluated at the time t_switch_ after pre-polarisation is switched off, is significantly higher for synchronised angular velocity.

Figure 5 shows signal strength after pre-polarisation is switched off for the two switching modes, normalised to the signal yield of an ideal transition. Signal strength for both switching modes decreases with longer switching time and higher **B**_**m**_. The expected MRI signal strength is significantly lower for switching with equal angular velocity.

**Figure 6:**
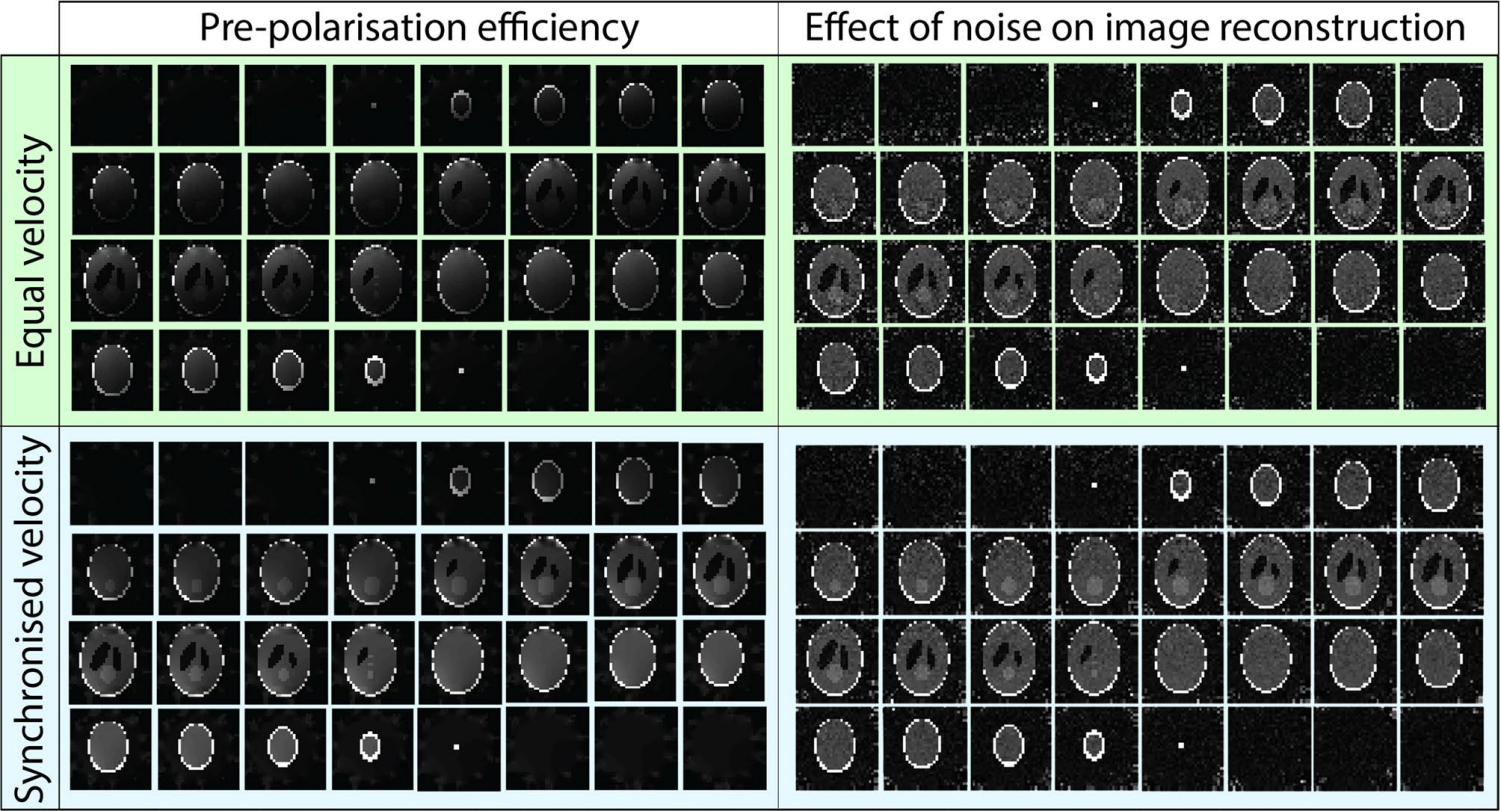
Visualisation of the averaged pre-polarisation efficiency of two non-adiabatic pre-polarisation removal strategies and the effect of noise (SNR ≈ 30) on simulated 3D 32 × 32 × 32 Shepp-Logan phantom image reconstruction. The higher MRI signal yield resulting from the synchronised velocity approach indicates a more efficient pre-polarisation cycle. The reconstructed images show more noise in the equal velocity **B**_**p**_ transition leading to a 32% higher error compared to the synchronised velocity transition. In both cases, noise is generally higher in regions where the efficiency of pre-polarisation is lower.

Both switching approaches show different pre-polarisation efficiencies through the averaged signal intensity visualised in Fig. 6. This efficiency is spatially dependent. Figure 6 also shows the effect that noise has on the reconstructed image for both cases. For a *t*_*switch*_ of 10 ms, the error in the reconstructed image is 32% higher with the equal velocity approach than with the synchronised velocity approach.

### Energy and magnetic force distribution

**Figure 7:**
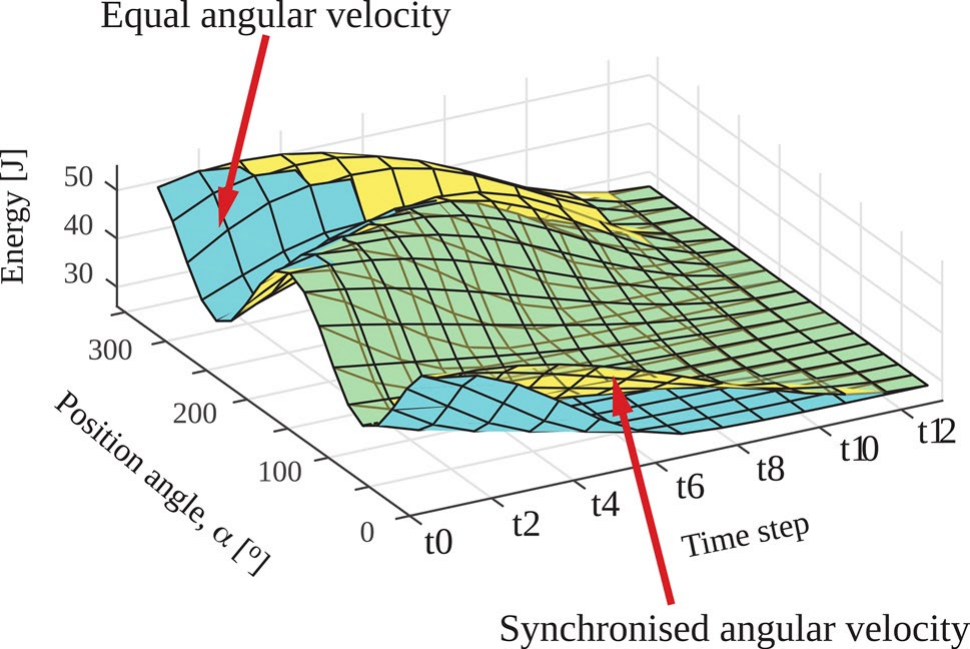
Energy distribution in the pre-polarisation array for synchronised and equal angular velocity. **B**_**p**_ is switched on at time step t_0_ and switched off at time step t_12_.

**Figure 8:**
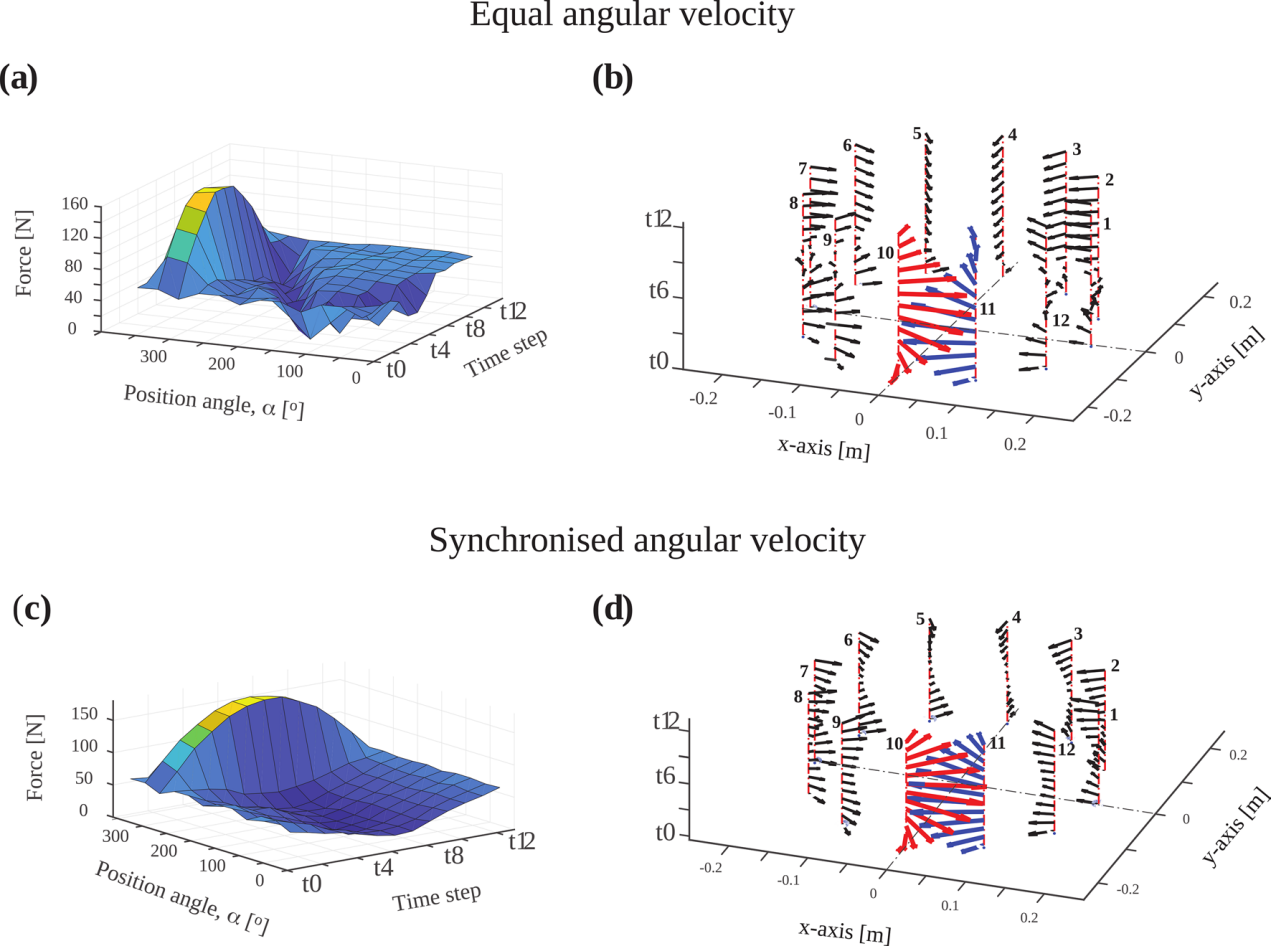
Magnetic forces during switching for an array with 12 magnet rungs. Distribution of (**a**) magnitude and (**b**) direction of magnetic forces for the switching mode with equal angular velocity. For magnets 10 (red arrows) and 11 (blue arrows), strong attracting forces between both magnets lead to the magnitude peak shown in (**a**). Distribution of (**c**) magnitude and (**d**) direction for location-dependent angular velocity. Only minor differences between switching modes, are observed (see also Table 2). Time step t_0_ corresponds to **B**_**p**_ switched on and t_12_ to **B**_**p**_ switched off.

Figure 7 displays the time varying magnetic energy in the pre-polarisation array as a surface plot from *t*_0_ (**B**_**p**_ switched on) to *t*_12_ (**B**_**p**_ switched off) with equal (blue surface) and location-dependent angular velocity (yellow surface). At *t*_0_, the magnetic energy is sinusoidally distributed around the circumference, while at *t*_12_, the energy is equally distributed among the magnets, as expected from our previous study^10^. Our result also indicated only minor differences between the two switching modes. There was little change in maximum stored magnetic energy as the number of magnet rungs increased from 12 to 20 (Table 2). Figure 8 shows the temporal evolution of the distribution of magnitude (Fig. 8a,c) and direction (Fig. 8b,d) of magnetic force in an array with 12 rungs, for equal (Fig. 8a) and location-dependent angular velocity (Fig. 8c). The peak force occurred at *α ≈ 3*π*/2* (270°) and exceeded the average force acting on the remaining magnets by ~ 300%. Differences between the two switching modes were minor.

**Table 2:** Maximum stored energy and maximum force for arrays with 12, 16 and 20 rungs.

Number of magnets	Centre field strength (mT)	Max energy (Joule)		Max. force (Newton)	
		Equal vel	Synch vel	Equal vel	Synch vel
12	52.8	42.93	43.12	159.57	161.41
16	69.2	46.04	46.22	384.26	388.08
20	85.7	50.51	50.82	755.56	762.05

### Mechanical stress within magnet rungs

The tangential component of the magnetic force (surface normal **n** ⊥ **F**_**mag**_) arising from the maximum estimated load of |**F**_**mag**_|= 762 N (see Table 2) resulted in a torque τ_**in**_ = 9.68 Nm. The torque τ_**in**_ during acceleration due to inertia was estimated using Newton’s second law for rotational motions with τ_**in**_ being equal to the angular acceleration multiplied by the moment of inertia. A switching time of 10 ms, with rapid deceleration of magnet rotation over 1 ms at the end of switching, were used in order to increase signal yield under non-adiabatic conditions. After acceleration to a constant angular velocity of ~ 628 rad/s and a deceleration rate of 6.28 × 10^5^ rad/s^2^, the maximum torque τ_**max**_ is less than 100 Nm.

**Figure 9:**
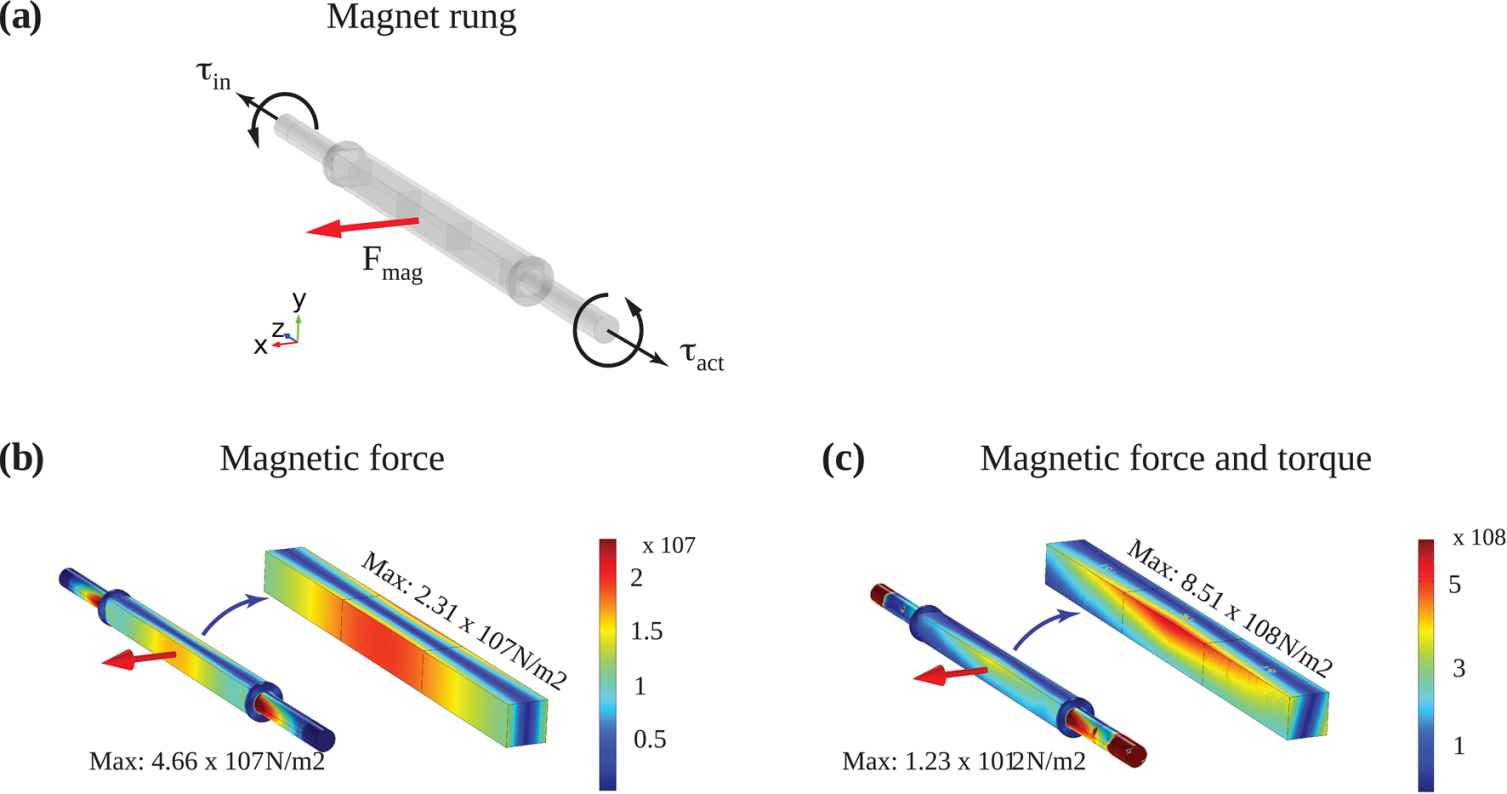
Visualisation of forces in a magnet rung. (**a**) Permanent magnet rung with two identical but opposite torques of τ_act_ = τ_in_ = 100 Nm on each end. (**b**) Von Mises stress for rung and permanent magnets with static magnetic force. The maximal rung stress is 4.66 × 10^7^ N/m^2^and 2.31 × 10^7^ N/m^2^ in the magnet. (**c**) Von Mises stress for magnetic force and applied torque. The maximum magnet stress is 8.51 × 10^8^ N/m^2^. In all cases, the maximum stress in the permanent magnets is three orders of magnitude lower than the shear modulus G.

**Figure 10:**
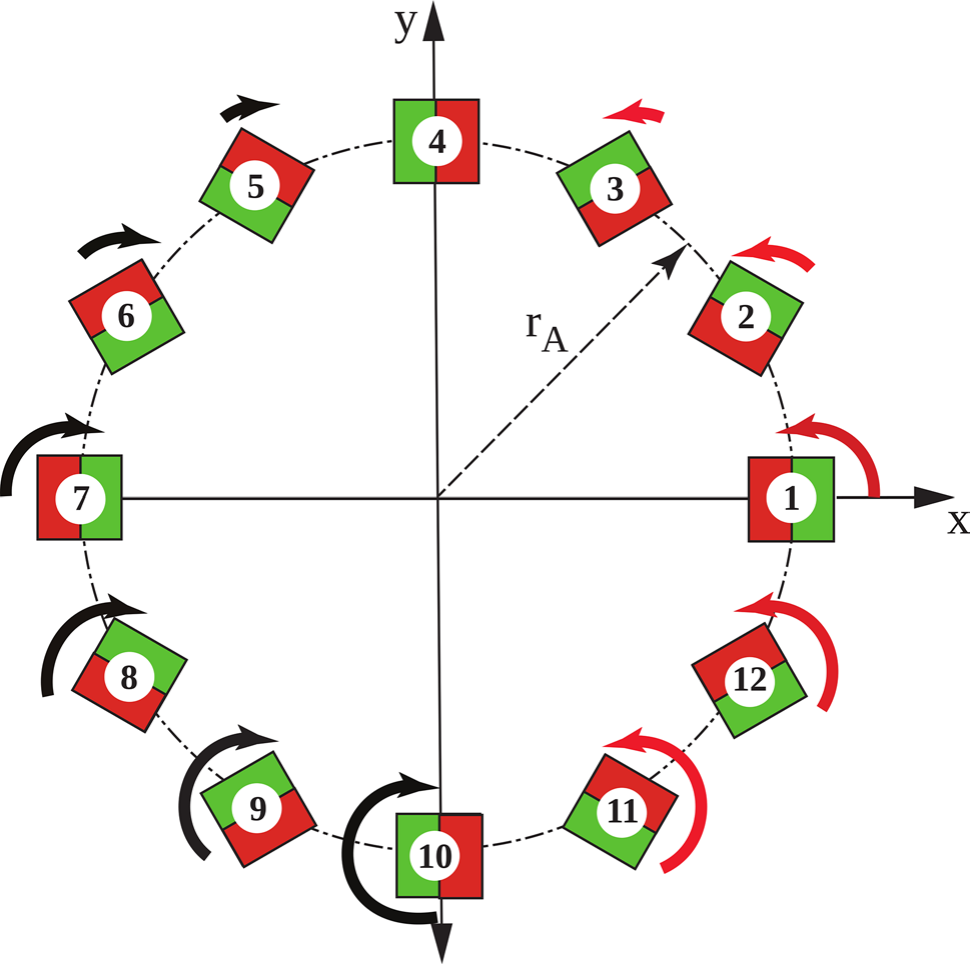
Direction of rotation of a pre-polarisation array with 12 magnet rungs. The arc length indicates the rotation angle required for switching from the Halbach to the tangential magnetisation pattern and the colour indicates the direction of rotation (black: clockwise, red: anti-clockwise). Rung 4 does not rotate. Rungs 10 and 11 rotate in opposite directions over the largest rotation angles.

Figure 9a illustrates the single magnet rung with the applied maximal force and the torques respectively, indicated by red and black arrows. Figure 9b shows the Von Mises stress distribution with a single load **F**_**mag**_ without a torque. Spatial regions of high stress are indicated by red, regions of low stress by blue with the COMSOL colour scheme *rainbow*. The maximal Von Mises stress is 4.66 × 10^7^ N/m^2^ for the rung. Figure 9c shows the Von Mises stress evaluation for magnet rung 10 with the maximal attraction or repellent force, |**F**_**mag**_|= 762 N and two opposing torques τ_**act**_ = -τ_**in**_ = 100 Nm (black arc arrow in Fig. 10a) applied at each magnet rung end. The maximum Von Mises stress is 8.51 × 10^8^ N/m^2^ for the permanent magnets. Our simulation also predicts higher stress levels (1.23 × 10^12^ N/m^2^) in the brass flange (part 1, Fig. 1b) compared to the magnet rung. This may require, for instance, larger axis diameter to reduce the mechanical stress. Since this is beyond the scope of this paper, we will not discuss it here in detail.

## Discussion

We analysed the magnetic field distribution, mechanical forces and magnetic energy during switching of the pre-polarisation magnetic field in an Ultra-low field MRI instrument based on permanent magnet arrays. Different patterns of magnet rotation to achieve field cancellation result in differences in the temporal evolution of the magnetic field to which an object in the field of view is exposed. We considered two switching modes, one in which all magnet rungs in the array rotate with equal angular velocity and one in which angular velocity varied with magnet location such that rotation stopped synchronously (Fig. 4). We showed that the expected MRI signal yield decreases with higher measurement field **B**_**m**_ and increased switching times *t*_*switch*_, as illustrated in Fig. 5. A more efficient non-adiabatic transition from **B**_**p**_ to **B**_**m**_ is achieved when the rotation of the resultant field orientation (**B**_**p**_ + **B**_**m**_) is much faster than the Larmor frequency (i.e., |d**B**_**p**_/dt|> >|γ**B**^2^_**m**_|), where γ is the gyromagnetic ratio)^24^. Here, the most critical stage determining the efficiency of the non-adiabatic transition is during the time that the magnitude of **B**_**p**_ and **B**_**m**_ are in the same order of magnitude. Therefore, a higher **B**_**m**_ requires a faster removal of **B**_**p**_ to sustain the non-adiabatic condition.

The pre-polarisation efficiency of 90% estimated with the synchronised velocity mode (*T*_*switch*_ = 10 ms, **B**_**p**_ = 48 mT, and **B**_**m**_ = 50 µT) increases NMR signal strength by almost 3 orders of magnitude compared to signal strength in the absence of pre-polarisation. Such a dramatic SNR increase justifies the efforts to develop efficient pre-polarisation methods.

As shown in Fig. 6, removing the pre-polarisation field by rotating the permanent magnets with an angular velocity that varies with magnet location such that rotation stopped synchronously yields higher simulated image quality. This transition strategy is also more robust against noise due to more efficient non-adiabatic transition from **B**_**p**_ to **B**_**m**_. Additionally, the effect of noise on the images has a strong spatial dependence because neither of the methods to remove **B**_**p**_ considered here resulted in homogeneous **B**_**p**_ field removal. This spatial dependence is stronger in the case of the equal velocity transition. Another factor contributing to the non-uniform distribution of noise is the overall lower encoding field gradient strength in regions further away from the encoding magnets. Reduced frequency separation between neighbouring voxels degrades the conditioning of the encoding matrix^25–27^. Regions experiencing weaker gradient fields are more susceptible to noise and are predominantly in the centre of the phantom due to its larger distance to the encoding satellite magnets. Our evaluation of dynamic energy and magnetic force distribution supports the feasibility of dynamic permanent magnet arrays for pre-polarisation in ULF MRI. When pre-polarisation is ‘switched off’, the magnetic energy is redistributed until equilibrium is reached, at which point each magnet has the same lowest possible magnetic energy^10^. The distribution of magnetic forces during switching is asymmetric and is highest where magnet rungs are counter-rotating over large angles and experience strong attracting forces. The change in the orientation of magnets from the Halbach to the tangential orientation necessitates adjacent counter-rotating magnets which results in a force peak.

Since magnet rung 10 experiences the strongest force and torques over the widest rotation angle (see Fig. 10), our stress analysis focussed on this magnet rung. We considered a static single load with one maximal force and a dynamic combined load of this force and two torques. Under both conditions, stresses in the magnet are more than three orders of magnitude lower than the shear modulus G of Neodymium (~ 10^11^ N/m^2^, see Table 1) giving us confidence that the integrity of the permanent magnets would not be threatened. Although this study focussed on the dynamic effects and mechanical stress during switching and imaging of dynamic permanent magnet arrays in ULF MRI, the simulation approach can be extended to study switching of quadrupole or even higher order cylindrical magnetic field distributions used, for instance, in particle physics.

## Data Availability

The datasets generated during and/or analysed during the current study are available from the corresponding author on reasonable request.
